# Physical assessments of termites (Termitidae) under 2.45 GHz microwave irradiation

**DOI:** 10.1038/s41598-020-61902-6

**Published:** 2020-03-23

**Authors:** Aya Yanagawa, Atsushi Kajiwara, Hiroki Nakajima, Elie Desmond-Le Quéméner, Jean-Philippe Steyer, Vernard Lewis, Tomohiko Mitani

**Affiliations:** 1 Research Institute for Sustainable Humanosphere, Kyoto University, Uji, 611-0011 Japan; 2 Nara University of Education, Takabatake-cho, Nara, 630-8528 Japan; 3 Department of Molecular Chemistry, Graduate School of Kyoto Institute of Technology, Kyoto, 606-8585 Japan; 4 INRAE, Univ Montpellier, LBE, Narbonne, France; 5 Department of Environmental Science, Policy, & Management University of California Berkeley, CA, 94720 USA

**Keywords:** Biophysics, Engineering

## Abstract

Demands for chemical-free treatments for controlling insect pests are increasing worldwide. One such treatment is microwave heating; however, two critical issues arise when using microwaves as a heat source: intensive labor and excessive energy-consumption. Optimization is thus required to reduce energy consumption while effectively killing insects. Currently, the lethal effect of microwaves on insects is considered to be due to the temperature of the irradiated materials. This study examines how the conditions of irradiation, such as resonance or traveling mode, changed the conversion of electromagnetic energy into heat when 2.45 GHz microwaves penetrated the body of the termite, *C. formosanus*. Our results indicated that it is possible to heat and kill termites with microwaves under resonance condition. Termites were however found to be very tolerant to microwave irradiation as the permittivity of the insect was low compared with other reported insects and plants. Electron spin resonance revealed that termites contained several paramagnetic substances in their bodies, such as Fe^3+^, Cu^2+^, Mn^2+^, and organic radicals. Interestingly, irradiation with traveling microwaves hardly produced heat, but increased the organic radicals in termite bodies indicating non-thermal effects of microwaves.

## Introduction

The use of electromagnetic waves such as microwaves and radio frequency waves (RF) for pest control through heating is not fundamentally new and was first tested and reported in the 1930s^1^. These authors reported a possible mechanism through body water contents and biological factors^2^; however, results were speculative. RF can penetrate non-metal substances efficiently, and thus, high-water content products like fruits tend to be treated with RF. Microwaves are also effective for producing heat energy in a short time and are thus applied to low-water content products such as rice or cereals^3–6^. Many reports support the use of microwaves in controlling pests including termites^7–14^. Although microwaves allow efficient and rapid heating deep inside treated materials, the high-energy consumption required for optimal results is a major drawback^15,16^. Microwave heating has been traditionally used in the spot control of dry wood termites as they are classified as a single-piece infester of timbers^17^. However, this treatment method has had mixed results and some damage to test boards was noted^10^.

Microwave heating has been characterized as a non-chemical method for insect control. Its mode-of-action is based on the dielectric heating and conversion of absorbed electromagnetic energy to thermal energy throughout the irradiated substrate^17^. An advantage of microwave irradiation is the rapid temperature increase in the interior of the irradiated material regardless of its thermal conduction properties. Energy consumption for heat treatments has the potential to be considerably reduced when using microwaves^4^. During the last decade, several studies have supported the efficiency of microwave heating of wood^18–25^ and wood-invading insects^7,10,11,26^. However, some other studies reported reduced or no lethal impact on pests^24,27,28^. According to Nakai *et al*.^27^, direct exposure of the termite body to microwave radiation did not show a lethal effect. The high temperature of the timber obtained through microwave irradiation killed wood pests^24,28^. To be lethal, the complete heating of the relevant material is required up to a minimum temperature of 55 °C for a duration of at least 60 min. Owing to the low heat conductivity of wood, the heating of its interior is relatively time intensive. However, in the treatment of agricultural products, pest insects generally die in much shorter time frame^2,29^. It may be possible that factors other than temperature increase insect death. The current microwave devices for heat-treated termite control are cumbersome, consume significant electric supply, and are therefore dangerous to use. Investigating the fundamental physical impact of microwaves on termites can help to improve device efficiency.

In this study, the objective was to determine the impact of 2.45 GHz microwaves on the termite body using a subterranean termite, *Coptotermes formosanus* Shiraki, one of the most destructive insects of houses and wood structures worldwide^30–32^. Transduction of electromagnetic energy to heat in termite bodies was evaluated for standing waves generated by resonance conditions and for traveling waves. Permittivity of the termites was measured and used to interpret the results of energy transduction experiments. Finally electron spin resonance (ESR) measurements were carried out to try identifying potential paramagnetic substances in termite bodies that interact with the electromagnetic field and to track changes in chemical composition linked with non-lethal irradiations.

## Results

### Direct exposure to microwave under resonance conditions (standing waves)

**Figure 1:**
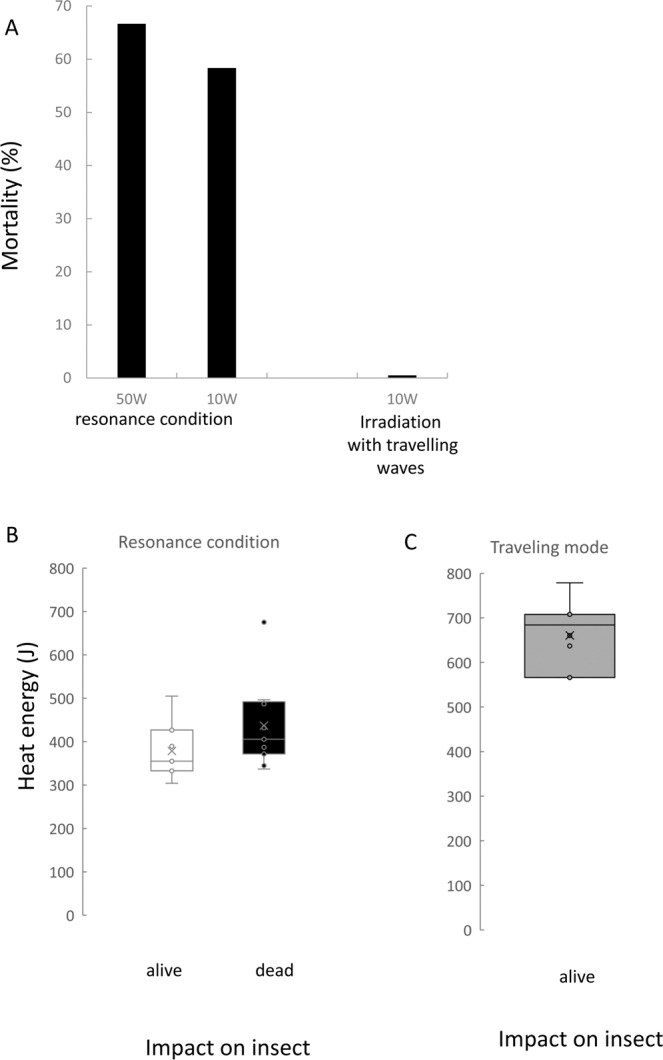
Patterns of microwave energy transduction in the insect body under two irradiation conditions. (**A**) Mortality of the termite under each microwave irradiation condition. (**B**) Energy absorption of an insect body under the irradiation with a resonating wave. (**C**) Energy absorption of 30 insects (80.5 mg) under the irradiation with traveling waves. Vertical bars represent standard errors (SE).

**Figure 2:**
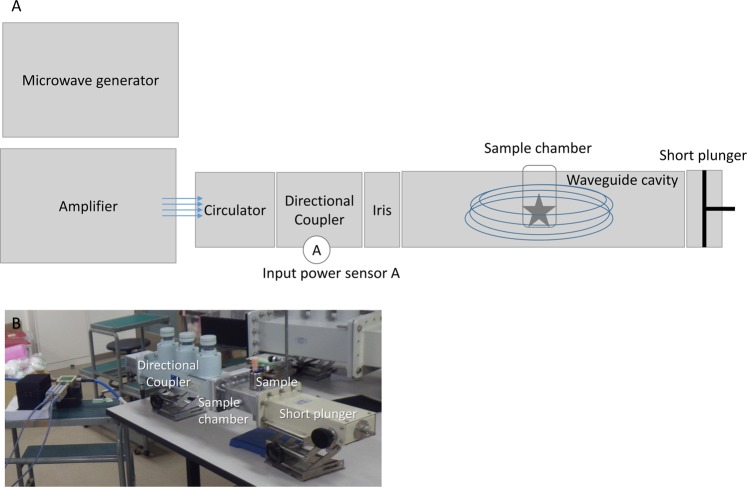
Microwave irradiation device to create resonance condition. (**A**) Device connection, (**B**) Device photo. Arrows indicate the microwave direction.

Under resonance conditions with single-mode microwaves, microwave energy was turned into heat energy inside the insect bodies As a result, 80–99% microwave energy was constantly absorbed into the insect body. 100% of sample insects stopped moving within 1 min and approximately 60% of them were dead after the exposure (Fig. 1A). The energy absorption of the insect bodies during the irradiation was calculated by the absorption rate and are shown in Fig. 1B. For example, for 80% energy absorption, where termites were irradiated with 50 W microwave energy (input power sensor A, Fig. 2) at the inflow monitor, the outflow monitor showed that 10 W of microwave energy (output power sensor B, Fig. 2) had passed through the device. It indicated that 40 W microwave energy, or 80% of the total, was transferred into heat energy throughout the insect body. Microwaves were irradiated into the sample termites until they stopped moving; this took approximately 10–60 s. The energy range required to kill a termite was 344.8–675.0 J. While individual variations were observed, the lethal threshold of heat energy absorbance appeared to exist between 378.3–436.7 J.

### Direct exposure to traveling microwaves

**Figure 3:**
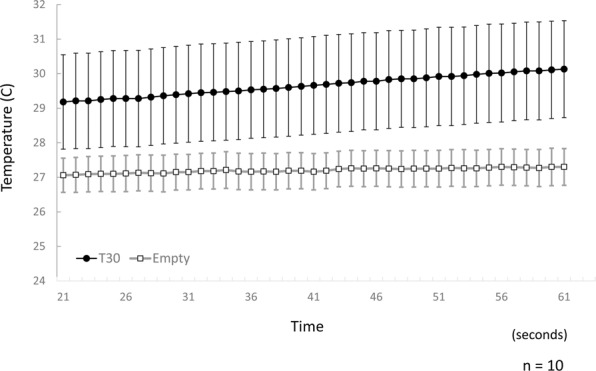
Temperature of arena during 10 measurements under microwave irradiation with traveling waves. Vertical bars represent standard errors (SE).

Microwave energy was converted into heat energy through the insect body although with low efficiency. The energy absorption of 30 insect bodies is described in Fig. 1C. No insect died after three minutes of irradiation (Fig. 1A). Because the termite body temperature increase was not stable during the initial moment of irradiation, the recorded energy transition ranged from 20–60 s (Fig. 3; Suppl. Data 1). The temperature of the petri dish with insects increased at a rate of: Tc_1_ = 0.0244 ± 0.0013 s, and the empty dish (control) increased at a rate of: Tc_2_ = 0.0050 ± 0.0007 s. Hence, the estimation of the energy absorbance rate of the termite body was: Tt = T_c1_ − T_c2_ = 0.0019 ± 0.0017 s, where Tc is the increase in chamber temperature, s is time in seconds, and Tt is the increase in termite body temperature.

The body heat-energy absorption of the insect body at 2.45 GHz was estimated using the specific heat of water 4180 J/(kg·K) and water content of the termite body (70%):

Power absorbance P_B_ = [0.0805(g) × 4.180 (specific heat of water in J/g/K) × 0.7 (body water content) × 0.7484 (temperature increase for 40 s in K)]/40(s) = 0.0044 W = 0.0044 J/s.

Absorbance rate (%) =100 (P_B_/P_A_) = 100 (0.0044/10) = 0.044%

(P_A_: irradiation power, P_B_: Power absorbance)

### Termite permittivity

Worker termite permittivity was measured on vials filled with termites; thus, the measurements varied with the amount of air in the vials. Permittivity was, therefore, adjusted using the calculation of Nelson *et al*.^33^. The result of the measurement of the air-insect mixture permittivity is described in Suppl. Data 2. The permittivity of the 2.45 GHz microwaves at 23 ± 3 °C averaged 14.83 ± 4 from 5 density (Suppl. Data 2A). Permittivity from the highest insect density was 18.17. Both values were adjusted according to Nelson’s calculation. The water content of the sample insects was measured at 69.8%.

Permittivity can indicate the penetration depth in the insect body^34^.

The average permittivity and Tanσ at 2.45 GHz (n = 10) from the above measurement without an offset process were taken for this calculation:

$$\begin{array}{rcl}Penetration\,depth & = & \frac{{\rm{c}}}{2{\rm{\pi }}{\rm{f}}(2{{\rm{\varepsilon }}{\prime} }_{r}{)}^{\frac{1}{2}}}{\left\{{\left[1+{\left(\frac{{{\rm{\varepsilon }}{\prime\prime} }_{r}}{{{\rm{\varepsilon }}{\prime} }_{r}}\right)}^{2}\right]}^{\frac{1}{2}}-1\right\}}^{-\frac{1}{2}}\\  & = & [2.998\times {10}^{8}]/2\surd 2\pi \ast 2.45\times {10}^{9}{\{5.088[\surd 1+{(0.097/5.088)}^{2}-1]\}}^{1/2}\\  & = & 4.53\,{\rm{m}}\end{array}$$

where f is frequency, ${{\rm{\varepsilon }}{\prime} }_{r}$ is the insect relative permittivity without Nelson’s offset calculation, ${{\rm{\varepsilon }}{\prime\prime} }_{r}$/${{\rm{\varepsilon }}{\prime} }_{r}$, dielectric loss)

### ESR measurements of termites

**Figure 4:**
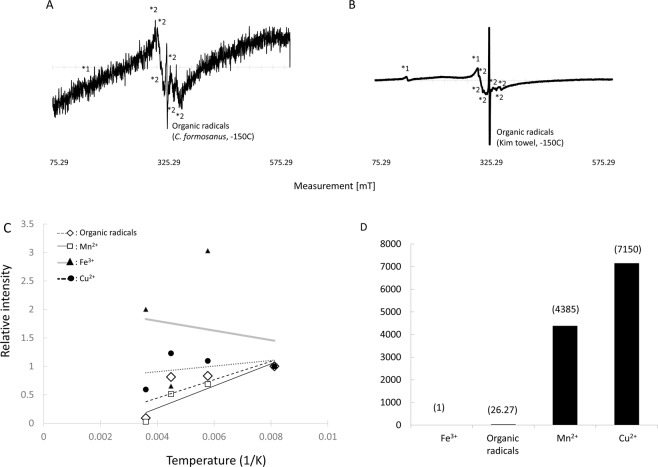
ESR spectra of the termite, *C. formosanus*. *1 indicated the spectrum of iron (Fe^3+^). *2 indicated the spectra of manganese (Mn^2+^). The S-shape curve over Mn spectra indicated the presence of cupper (Cu^2+^). (**A**) ESR spectrum of the termite, *C. formosanus* (34.5 mg). (**B**) ESR spectrum of kim towel (control, 47.9 mg). (**C**) Interactions with microwave suggested by ESR spectrum. If the relative intensity increased proportionally, it suggested that there was no interaction with microwaves. The intensity at −150 °C was set as standard (=1) for each paramagnetic substance to compare the intensity with other temperatures. (**K**) temperature in kelvin unit, (**D**) Relative intensity of paramagnetic substances at −150 °C when Fe^3+^ was set as standard (=1) to compare the intensity with other paramagnetic substances. The spectra at −166 was used as measurement was at this temperature.

Figure 4 shows the ESR spectrum of termite *C. formosanus*. ESR revealed that pseudergates contained several paramagnetic substances in their bodies such as Fe^3+^, Cu^2+^, Mn^2+^, and organic radicals (Fig. 4A). Kim towel, the control material, also possessed Fe^3+^ and organic radicals (Fig. 4B). All those paramagnetic substances existed in their native food source, *Pinus* sp. (Suppl. Data 2). Relative intensity was measured using the spectrum area value at each temperature to observe the interactions with microwaves on each paramagnetic substance (Fig. 4C). Temperature-dependent signal intensity suggested the interaction of these paramagnetic substances with other molecules. Had it shown a proportionately-shaped increase, the substance existed independently in the termite body; if it had combined with other molecules, the interaction between these molecules changed the pattern. Hence, if the temperature-dependent intensity did not show the proportional increase, it suggested that some proteins or peptides combined to the substance in the microwave-sensitive structure. In termites, except Mn^2+^, all other detected paramagnetic substances affected the physiological mechanism. Though Fe^3+^ showed the greatest interaction the with microwaves (Fig. 4C), the relative intensity indicated that the most dominant paramagnetic substance in *C. formosanus* was Cu^2+^, while Fe^3+^ as the least dominant (Fig. 4D).

**Figure 5:**
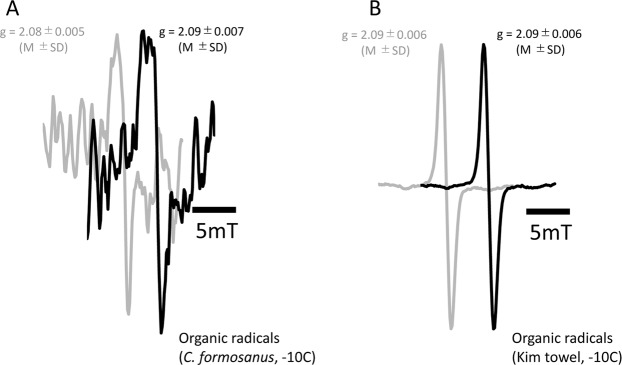
ESR spectra of organic radicals under 2.45 GHz microwave irradiation. g-value indicates the dimensionless quantity that characterized the magnetic moment and angular momentum of a composite particle, a particle, or nucleus. (**A**) ESR spectra of organic radicals showing the increase of free radicals in the termite, *C. formosanus* (815.2 mg) under the microwave exposure of 2.45 GHz at −8.5 dBm (black) and 0 dBm (control, grey) (**B**) ESR spectra of organic radials showing no increase of free radicals in the control substance, kim towel (47.9 mg) under the microwave exposure of 2.45 GHz at −8.5 dBm (black) and at 0 dBm (control, grey).

The ESR spectrum during additional irradiation of 2.45 GHz microwaves revealed that organic radicals were produced by the microwave irradiation (Fig. 5A). Though the radical intensity was generally stronger at lower temperatures with ESR, the increase in free radicals was only possible to detect over −10 °C; hence, only the intensity of organic radicals was detectable. The relative intensity of organic radicals increased 2.32 times more than that of the control without irradiation (Fig. 5A). The increase in organic radicals did not occur in the control material (Kim towel fed to termites) (Fig. 5B) (relative intensity under microwave irradiation was 1.06). This increase appeared with the change in the biological interactions at the molecular level because the g-values of the termites had also shifted along with the increase in organic radicals.

## Discussions

In this study, to clarify and further our basic understanding of how microwaves interact with the internal physiological mechanisms of termites *C. formosanus*, we measured the energy absorption of the insect body under two different irradiation conditions. The results indicated that the efficiency of transduction of electromagnetic energy to heat depended on the irradiation condition. The resonating microwave was dramatically more efficient in producing heat energy. Consequently, termite death was caused by the heat. Although the energy of traveling microwaves was absorbed into insects, the energy transition efficiency from microwave energy to heat energy was only 0.044%. Thus, its irradiation was not lethal to the insect. The permittivity of termites is low, while ESR showed that termites contain several paramagnetic substances in the body such as Fe^3+^, Cu^2+^, Mn^2+^, and organic radicals. Finally, ESR under microwave irradiation revealed that traveling waves induced an increase in organic radicals in the insect bodies.

It is noteworthy that microwave energy was absorbed into the termite body as heat energy under resonance, in other words, standing wave conditions, but not under traveling wave conditions (Fig. 1). That is, microwave energy of traveling waves was easily released by radiation, and was not absorbed into the termite body; consequently, even a high-power irradiation by traveling waves would simply pass through the insect body. This can be explained by termite body size^27^ or its permittivity. The termite body consists of 70% water, similar to other beetles^33^; however, water, which has the same volume as the termite, could not absorb microwave energy effectively^27^. The permittivity of *C. formosanus* at 2.45 GHz was 14.83 (Suppl. Data 3A). Those of other wood pest beetles are generally in a range of 10–40^35,36^. Termite permittivity, therefore, is relatively low. As for termite body components, according to Itakura *et al*.^37^, the dry weight of worker termites is composed of 46.5% lipid, which has a low conductivity, and it is possible that termite body composition does not allow storage of electrical energy inside the body. The results also supported the finding that the energy efficiency of the methods used in this study strongly depends on the conditions of the transfer of the electromagnetic energy to the load represented by the volume to be treated.

The increase in free radicals detected by ESR was unexpected because microwaves are not an ionizing radiation such as ultraviolet (FUV) or X-ray^38^. Sterile insects are produced by the irradiation of ionizing radiation, whose treatment is also known to induce free radicals^39^. As for non-ionizing radiation, near-ultraviolet (NUV) such as UV-A in sunlight is known to induce free radicals and skin cancer, and UV-C is known to sterilize microbes. The increase of organic radicals in the present study suggested that some physical reactions were induced in the termite body during the microwave irradiation, producing free radicals. It also suggested that microwave irradiation, regardless of its strong power, does not have any lethal impact, but could induce similar physical damage as a pest sterilizer. Representative free radicals in living organisms are from oxidative stress and nitrosative stress, which are considered to play an important role in many human diseases^40,41^. The increase in free radicals was, however, not necessarily a sign of cellular damage because free radicals are also produced by general cell activities^40,42^. The g-value indicated the magnetic dipole component and the value was shifted (Fig. 5). This meant that the status of a magnetic moment and angular momentum in the termite body was changed by the microwave irradiation. ESR and previous experiments support the presence of magnetic-field-sensitive substances in the termite body^43^. It can simply mean that the microwave irradiation triggered some biological/physical reactions^40,42^.

ESR revealed that this termite possessed paramagnetic substances, Fe^3+^, Cu^2+^, and Mn^2+^. *C. formosanus* has a strong association with soil minerals^44^. The detected paramagnetic substances were similar to those found in ants, *Pachycondyla marginata*^45^. Spectra from the fed Kim towel were different from the one for the *C. formosanus* (Fig. 5), but similar to the original food source, *Pinus sp*. (Suppl. Data 3). Biochemical reaction base investigations are required to clarify the interactions of these substances with microwaves as there is a wide range of possible chemical association for each paramagnetic substance in the whole insect body. Generally, these paramagnetic minerals are important both for nutrition and sensory functions^46^. There are some reports supporting the idea that termites can sense the electromagnetic field of the earth^47–49^, although the underlying mechanism is unknown. Learning more about these paramagnetic substances may help us to find them.

Termite control by microwave heating currently relies on the heating of the wood around them^26,28,50^, similar to other heat treatments for other pests^51^. Interestingly, we demonstrated here that *C. formosanus* possess physical components that interact with microwaves, and current results support the possibility of improved efficiency in non-thermal treatments. To attain this goal, it would be crucial to understand interactions among the organic components, paramagnetic substances, and water in the insect. Clarifying the physiological mechanisms caused by microwave irradiation in insects will contribute to improved pest control systems using such irradiation.

## Materials and Methods

### Insects

*C. formosanus* were obtained from a laboratory colony B (Wakayama, Colony was established in the lab in 2015) and were maintained in the dark at a relative humidity of 85% and temperature of 28 °C (Deterioration Organism Laboratory (DOL) of the Research Institute for Sustainable Humanosphere, Kyoto University, Japan). We used only pseudergate termite workers for all experiments. Pseudergate workers were collected from stock colonies 1–2 weeks before the experiments and maintained in Petri dishes (90 × 15 mm), containing Kim towels that were moisten with distilled water.

### Direct exposure to microwave under resonance condition (standing waves)

To learn the mechanism of the microwave irradiation, pseudergates were exposed to single-mode microwaves in resonance conditions. The microwaves were generated from a solid-state amplifier (R&K, GA0827-4754-R) with a microwave oscillator (Agilent, MXG wave generator, N5183A) at Kyoto University: Analysis and Development System for Advanced Materials, consisting of a waveguide cavity, a 3-stub, and a short plunger (Fig. 2). 2.45 GHz microwaves were focused by an iris and formed a TE103 mode in this cavity. The iris had a 28-mm slit that was parallel to the direction of the electric field. The electromagnetic field was calculated by a finite element method (Software, FEMTET). During microwave irradiation, the profiles of irradiated inflowing microwave power and its outward flowing power were continuously monitored with a microwave power meter (Agilent N8485A).

A single termite at a time (≈0.002 g) was put into a glass tube and held down. Each tested insect was exposed to microwaves for 3 min or until motion ceased. Microwave energy absorbance was measured with two different generated powers, one 50 W (n = 6) and the other 10 W (n = 12).

### Direct exposure to traveling microwaves

**Figure 6:**
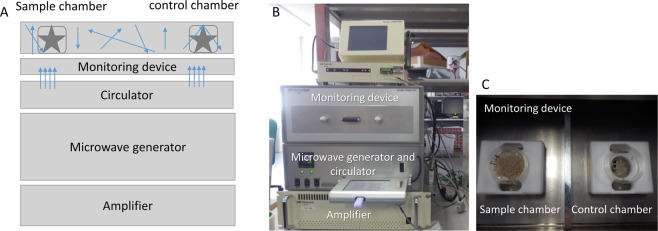
Travelling wave irradiation device with reflection. (**A**) Device connection, (**B**) Device photo, (**C**) photo of samples in monitoring device. Arrows indicate the microwave direction.

To determine the influence of ordinal microwave irradiation on the termites, the worker termites were exposed to traveling microwaves. A microwave irradiation system (Sunny Engineering Co., Ltd, MTS03(S), Osaka, Japan), consisting of a semiconductor microwave oscillator and an applicator, was used for the microwave irradiation experiments (Fig. 6). The semiconductor oscillator generated microwaves at 10 W and at a single and sharp frequency spectrum of 2.45 GHz. This device did not possess the sensors to monitor the inflow/outflow power of the energy but could monitor the temperature inside the applicator and the petri dish (3.5 cm diameter) with a thermistor. The dish material was Polystyrene, which interacted with the microwaves in nearly the same manner as the air^52^. Thus, the heat generated by the microwave irradiation efficiently occurred in the insect body rather than in the dish.

Worker termites were lightly anesthetized by placing them on ice to minimize their movement. Thirty termites (≈80.5 mg), just enough number to cover the bottom of the petri dish, were then collected and placed on the petri dish.

### Termite permittivity

Permittivity indicated the quantity of electric energy that materials could hold because it affected characteristic impedance. Therefore, termite permittivity was measured at 23 ± 3 °C using the method in Nelson *et al*.^33^.

Measurements of the relative complex permittivity, which is the dielectric loss factor, were made with a Keysight N1501A open-ended coaxial-line probe (N1501A-102, Keysight, USA) and an N5242A Network Analyzer (Agilent technologies, Japan). Permittivity measurements were conducted on five different densities of worker termites, 0.71, 0.81, 0.96, 1.15, and 1.43 g/cm^3^ (5 densities x 10 repetitions). Insects were collected in a polystyrene vial (4-1024-03 As One, Japan). As water is an important factor in permittivity, moisture content of the insects was determined by weighing them before and after drying. The insects were dried at 60 °C for 72 h.

### Paramagnetic components in termites

Electron Spin Resonance (ESR) detects the presence of unpaired electrons and allows one to visualize the free radicals in a substance directly and specifically. Thus, ESR spectra indicated the paramagnetic substance and g-value of the studied material. The g-value indicated the magnetic dipole component.

The ESR spectra of the radicals were recorded on a JEOL JES RE-2X spectrometer operating in the X-band, utilizing a 100 kHz field modulation, and a microwave power of 1 mW. A TE011 mode cavity was used. Temperature was controlled by a JEOL DVT2 variable temperature accessory. ESR measurements were performed at −5, −50, −100, and −150 °C. Spectroscopic simulations were carried out with a JEOL IPRIT Data Analysis System (version 6.4, JOEL Ltd, Japan) and spectra were analyzed. In addition, an additional portable microwave irradiation system was set up. ESR spectrum was also recorded under 2.45 GHz microwaves from this portable irradiation system. It was set on a mobile rack and consisted of a phased array antenna with circular-polarized antenna elements, a 2.45 GHz microwaves semiconductor generator, and amplifiers (total maximum power, 50 W) with a computerized beam control unit. An irradiative power of −8.5 dBm, was used, equivalent to approximately 19.5 mW/cm^2^ irradiation (Suppl. Data 4).

Worker termites were lightly anesthetized by placing them on ice and then transferred to the sample holder (JOEL DATUM ESR No:193 #422000281). As a control, ESR spectrum was also obtained from the Kim towel, which was fed to the termites for two weeks before recording to remove any plant materials from their alimentary tracts.

## Conclusions

This study showed that the energy transition efficiency from microwave energy to heat energy in insect body was only 0.044% with traveling waves but it becomes considerably high with resonating waves. This is probably because of their low permittivity. While ESR revealed that termites contained several paramagnetic substances such as Fe^3+^, Cu^2+^, Mn^2+^ and organic radicals. Irradiation of microwaves seems to affect insects physiologically due to these substances except Mn^2+^. ESR also showed that free radicals were induced in insect body by irradiation of traveling waves.

## Supplementary information

Supplementary Dataset 1,2,3,4.
