# Supplementary material for: Physical assessments of termites (Termitidae) under 2.45 GHz microwave irradiation
Source: Sci Rep. 2020 Mar 23;10:5197. doi: 10.1038/s41598-020-61902-6 (PMC7090069; doi:10.1038/s41598-020-61902-6)
Supplement: Supplementary file 1 — Supplementary Dataset 1,2,3,4. [file 41598_2020_61902_MOESM1_ESM.pdf]

Physical assessments of termites (Termitidae) under 2.45 GHz microwave irradiation

Aya Yanagawa, Atsushi Kajiwara, Hiroki Nakajima, Elie Desmond-Le Quemener, Jean-Philippe Steyer, Vernard Lewis, Tomohiko Mitani

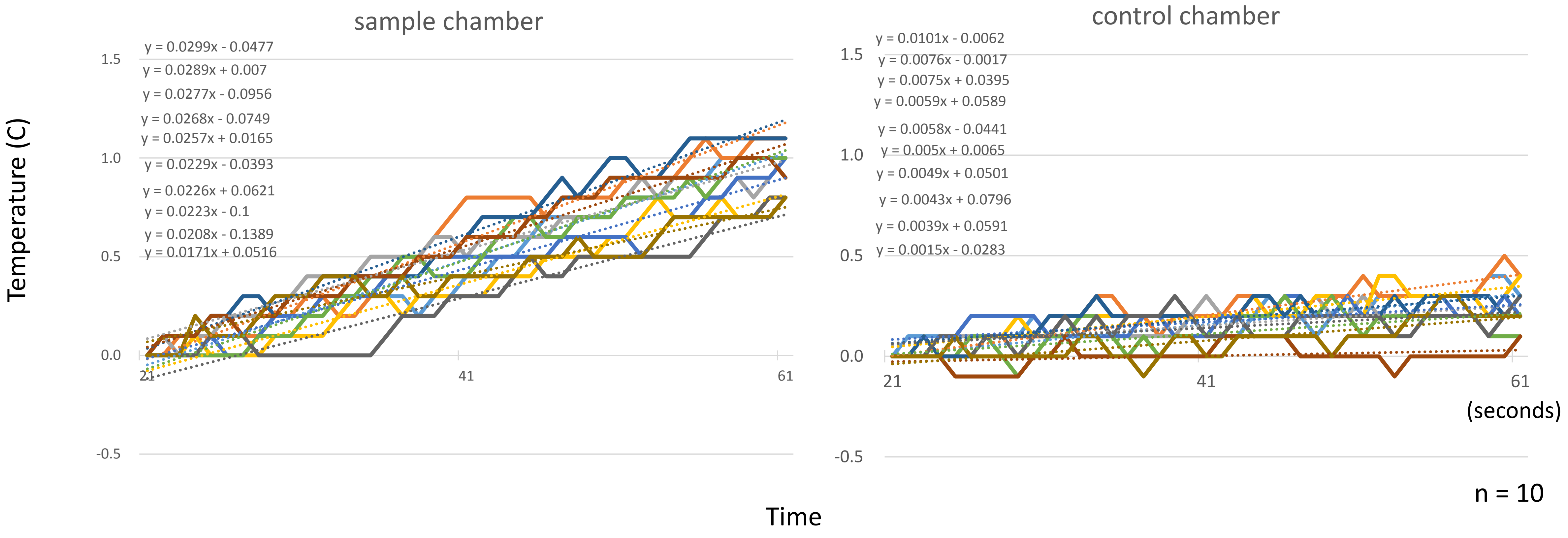

Suppl. Data 1. Temperature increase of 10 measurements of the termite, *C. formosanus* under microwave irradiation with traveling waves.

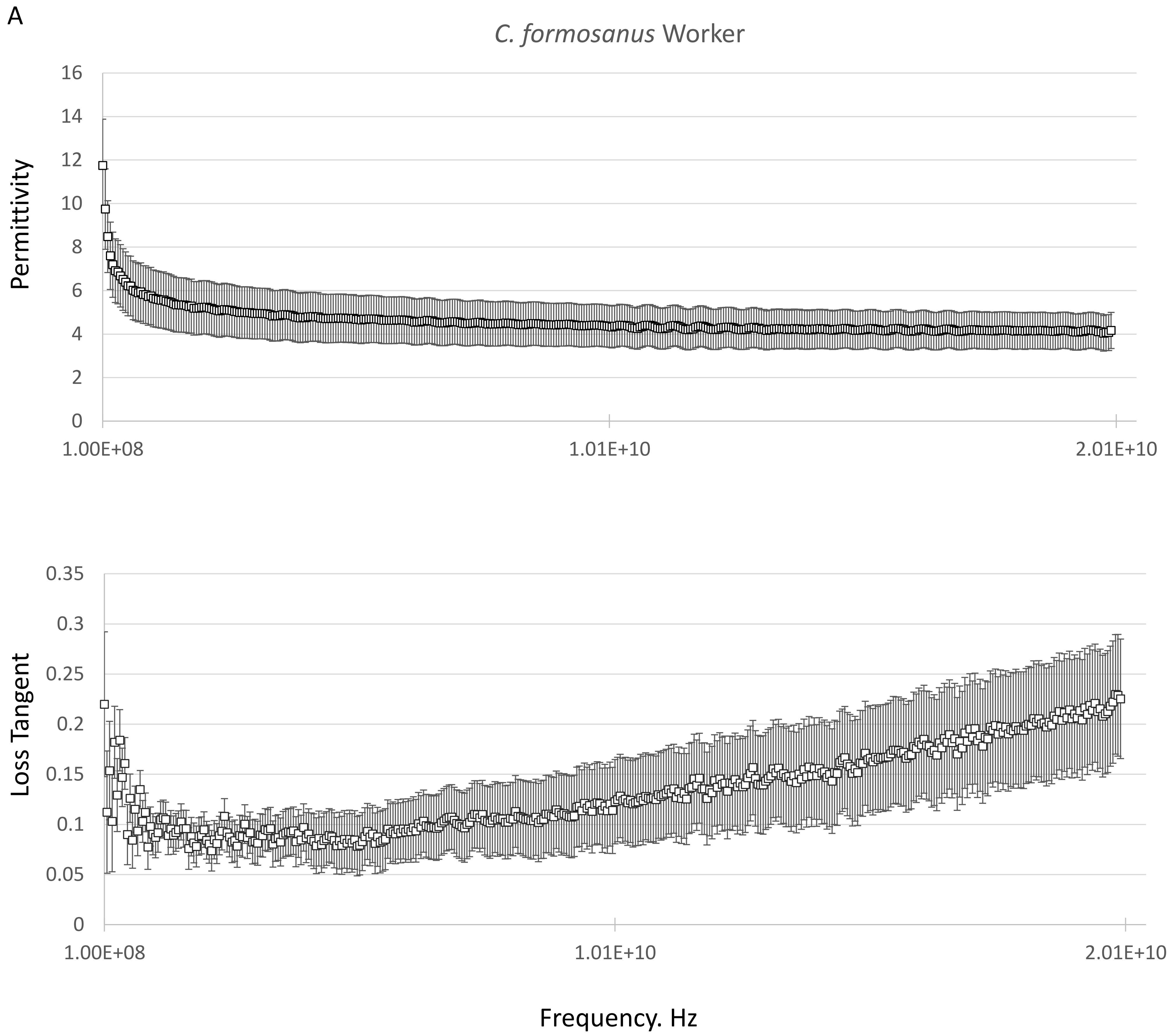

Suppl. Data 2.
A: Permittivity of the workers of the termite, *C. formosanus*.

B

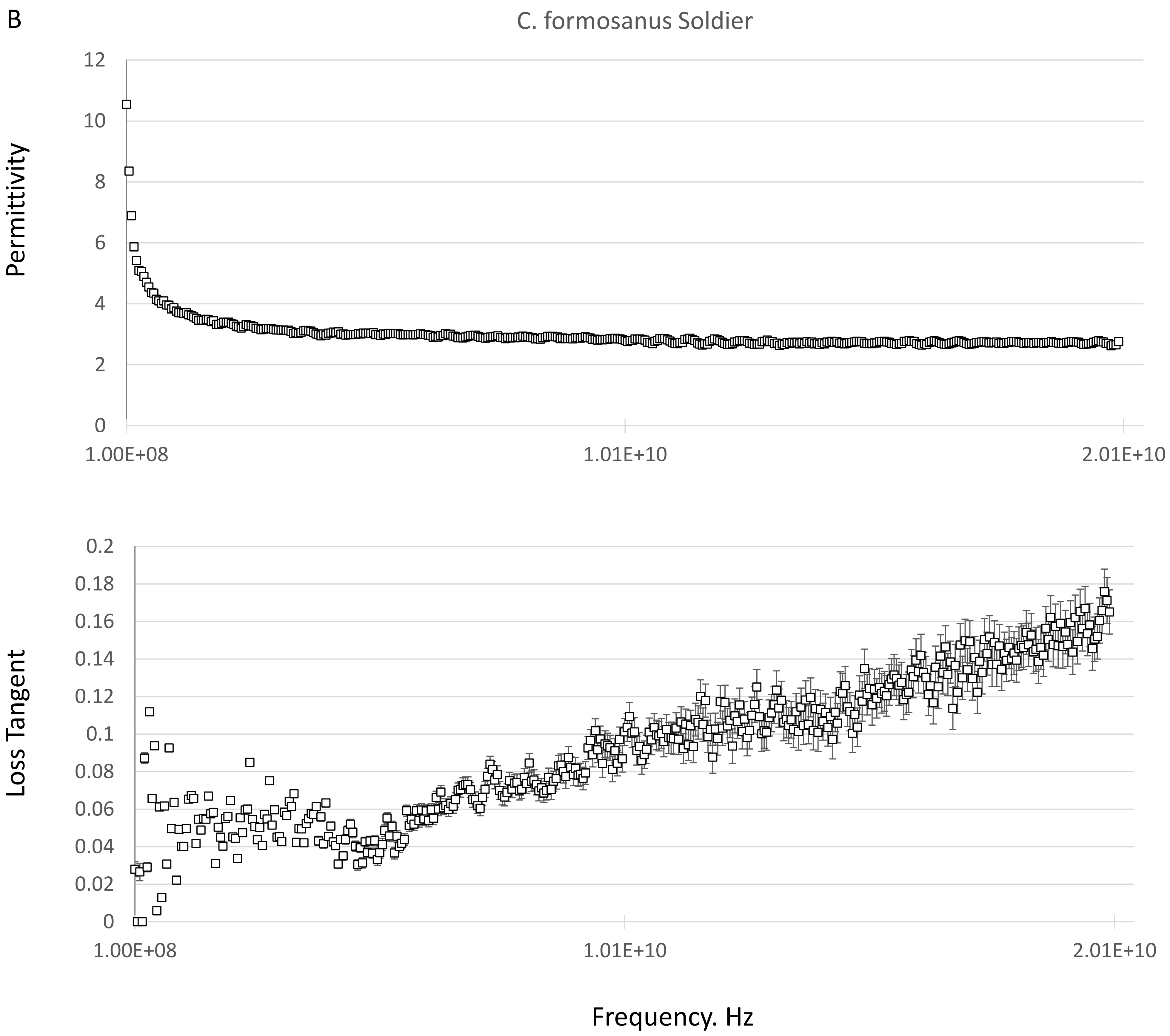

Suppl. Data 2.
B: Permittivity of the soldiers of the termite, *C. formosanus*.

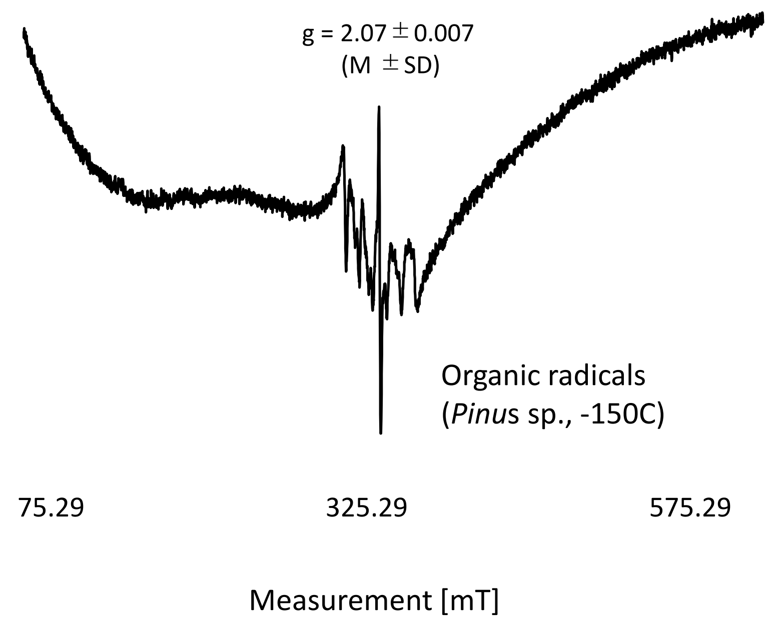

Suppl. Data 3. ESR spectrum of *Pinus sp*. (56.4 mg).

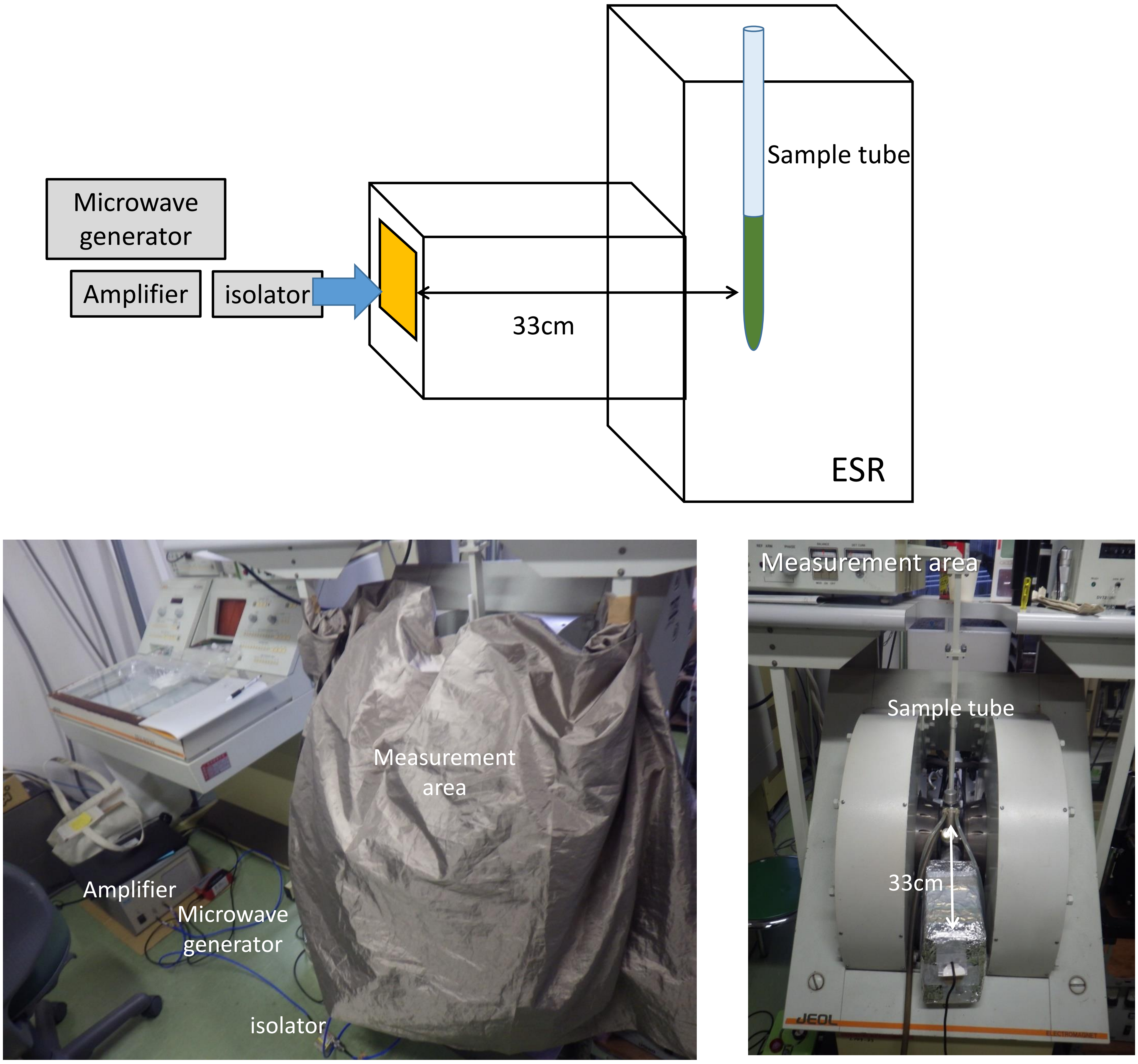

Suppl. Data 4. Portable traveling wave irradiation device to give the additional irradiation during ESR recording. Blue arrows indicate the microwave direction. Green part indicates the sample arena.
